# An Effective GNSS/PDR Fusion Positioning Algorithm on Smartphones for Challenging Scenarios

**DOI:** 10.3390/s24051452

**Published:** 2024-02-23

**Authors:** Jingkui Zhang, Baoguo Yu, Yuxiang Ge, Jingxiang Gao, Chuanzhen Sheng

**Affiliations:** 1 State Key Laboratory of Satellite Navigation System and Equipment Technology, Shijiazhuang 050081, China; lb18160005@cumt.edu.cn (J.Z.); cepntoa@163.com (B.Y.); shengchuanzhen@163.com (C.S.); 2 The 54th Research Institute of China Electronics Technology Group Corporation, Shijiazhuang 050081, China; 3 School of Environment and Spatial Informatics, China University of Mining and Technology, Xuzhou 221116, China; jxgao@cumt.edu.cn

**Keywords:** seamless indoor/outdoor positioning, GNSS/PDR fusion positioning, smartphone, real-time positioning, urban informatization and smart cities

## Abstract

The location-based smartphone service brings new development opportunities for seamless indoor/outdoor positioning. However, in complex scenarios such as cities, tunnels, overpasses, forests, etc., using only GNSS on smartphones cannot provide stable and reliable positioning results. Usually, additional sensors are needed to assist GNSS. This paper investigates the GNSS positioning algorithm assisted by pedestrian dead reckoning (PDR) in complex scenarios. First, we introduce a step detection algorithm based on the peak–valley of acceleration modulus, and the Weinberg model and the Mahony algorithm in PDR are used to estimate step length and heading. On this basis, we evaluated the performance of GNSS/PDR fusion positioning in an open scenario, a semiopen scenario, and a blocked scenario, respectively. Finally, we develop a GNSS/PDR real-time positioning software, called China University of Mining and Technology-POSitioning (CUMT-POS) version 1.0, on the Android 10 platform. By comparing GNSS solutions, PDR solutions, GNSS/PDR solutions, and real-time kinematic (RTK) solutions, we verify the potential auxiliary ability of PDR for GNSS positioning in complex environments, proving that multisource sensor fusion positioning significantly improves reliability and stability. Our research can help the realization of urban informatization and smart cities.

## 1. Introduction

The Global Navigation Satellite System (GNSS) can provide users with positioning, navigation, and timing services, and has been widely used in transportation, resource exploration, disaster prediction, aerospace, and military [1,2,3,4]. However, these positioning tasks usually require professional technicians and high-accuracy, high-cost GNSS receivers to complete, which seriously restricts the further applications of GNSS. In recent years, with the development of low-cost micro-electro-mechanical systems (MEMSs) and GNSS chips, more and more smartphones, tablet computers, smartwatches, and other portable mobile terminals have begun to provide positioning services. Among them, smartphones, which integrate communication, entertainment, work, and travel functions, have become indispensable devices in daily life.

In the early stages of positioning based on smartphones, the built-in module directly outputs information such as location and velocity, without providing a port to release the raw GNSS observations [5,6]. For technicians, the smartphone’s navigation and location function is a “black box”, so it cannot be further studied and analyzed. In 2016, Google announced that smart terminals equipped with Android Nougat 7.0 and above could output GNSS observations such as pseudorange and carrier phase measurements, which provides an opportunity for relevant technicians and engineers to conduct positioning algorithm research. With the rise of technological concepts such as 5G communication, the Internet of Things, and smart cities, people have increasingly high accuracy requirements for location services provided by smartphones. However, smartphones are usually only equipped with poorly performing polarized GNSS antennas and low-cost positioning chips. In the case of limited observation conditions, only GNSS may bring the problem of low positioning accuracy or even being unable to position itself [7,8].

Fortunately, smartphones are usually equipped with built-in accelerometers, gyroscopes, magnetometers, WiFi, Bluetooth, barometers, and so on. Using multisensor data to fuse with GNSS can solve the problem of positioning instability caused by GNSS signal interruption in complex environments, and can improve positioning accuracy significantly [9,10,11,12]. In the outdoor scenarios, fusion positioning based on GNSS and PDR has also been widely studied and applied to smart terminals. Recently, Jiang et al. [13] proposed an optimized particle filter method using the krill herd algorithm (KHA) for GNSS/PDR integration, significantly improving the integration positioning accuracy. Zhu et al. [14] analyzed the error characteristics of the inertial measurement unit (IMU) of smartphones and designed an improved GNSS/PDR fusion positioning method. The static horizontal positioning accuracy can reach the submeter level, while the dynamic horizontal positioning accuracy is about 2 m. Wu et al. [15] proposed to fuse the IMU data of the smartphone with the double-differenced pseudorange, which significantly improved the accuracy and robustness of the smartphone positioning. Jiang et al. [16] proposed a PDR/GNSS collaborative integration method based on factor graph optimization. A factor graph was constructed to represent the relationship between the status, measurement, and distance information of multiple agents, significantly improving the positioning accuracy under GNSS signal challenging conditions. In addition, Zhang et al. [17] analyzed the positioning performance of the GNSS/PDR fusion algorithm on smartphones in complex environments. Yan et al. [18] designed an improved Kalman filter based on smartphone GNSS and IMU data with different sampling frequencies. To solve the problem of improper handling of errors in GNSS/IMU fusion, Yang et al. [19] proposed an improved nonholonomic robust adaptive Kalman filter and the results demonstrated the improved accuracy. Similarly, Sun et al. [20] proposed a motion-model-assisted fusion algorithm based on GNSS/MEMS that detected gross errors through a constant yaw rate and velocity model and the chi-square test. Moreover, Jiang et al. [21] realized the GNSS/PDR fusion positioning based on Kalman filter and graph optimization, finding that graph optimization can significantly improve positioning accuracy. The above studies have shown that the use of fusion positioning in complex urban environments is very helpful in improving the stability and reliability of positioning results. However, most of the current research on the fusion positioning of smartphones is post-processing, so the real-time fusion positioning algorithm of smartphones needs to be further studied.

Among many positioning technologies carried by smartphones, PDR detects the step and estimates step length through the characteristics of acceleration data, and then calculates the position in combination with the heading angle. It has the advantages of simple implementation, low cost, and high accuracy. In this work, we first study the step detection, step length estimation, and heading estimation algorithms of PDR, and further analyze the performance of GNSS/PDR fusion positioning in an open environment, a semiopen environment, and a blocked environment. On this basis, we developed a real-time positioning software called CUMT-POS (China University of Mining and Technology-POSitioning) version 1.0, which integrates GNSS/PDR data of smartphones. Based on CUMT-POS version 1.0, we evaluated the performance of real-time GNSS/PDR fusion positioning. Our research helps solve the problems of non-real-time and unstable smartphone positioning in satellite signal occlusion environments such as tunnels and overpasses.

## 2. PDR Algorithm

### 2.1. Principles of PDR

**Figure 1:**
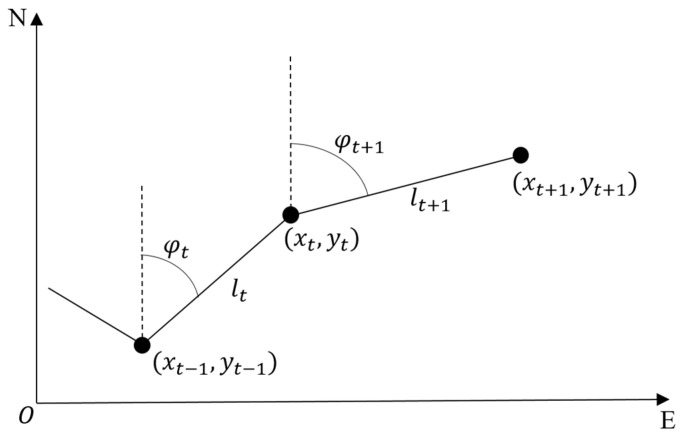
Positioning principles of PDR.

When a pedestrian walks, his/her body presents dynamic characteristics. PDR uses these characteristics to estimate the pedestrian’s gait information and then calculates the current position based on the previous position. The positioning principle of PDR is shown in Figure 1. Assuming that the pedestrian’s position at time *t* − 1 is (*x_t_*_−1_, *y_t_*_−1_), the pedestrian has moved a distance *l_t_* with a heading angle *φ_t_* from *t* − 1 to *t*. Then the pedestrian’s position (*x_t_*, *y_t_*) at time *t* can be calculated as follows:

(1) $$\left\{\begin{array}{l} x_{t}=x_{t-1}+l_{t}\sin\varphi_{t}\\ y_{t}=y_{t-1}+l_{t}\cos\varphi_{t} \end{array}\right.$$

Step detection, step length estimation, and heading estimation are the three parts of the PDR algorithm. We will introduce them in Section 2.2, Section 2.3 and Section 2.4, respectively.

### 2.2. Step Detection

When a pedestrian takes a step each time, the body will generate forward acceleration in the same direction, while the left or right foot will generate lateral acceleration. At the same time, as Combettes et al. [22] found, the up-and-down movement of the body will generate acceleration in the vertical direction due to gravity, resulting in obvious amplitude changes in the vertical direction. Therefore, the acceleration modulus will show periodic changes in peaks and valleys as the gait changes. Based on this characteristic, the step detection algorithm determines that the pedestrian takes a step each time when it detects a period of change. However, the modulus calculated from the original acceleration data usually contains a large amount of noise, which is manifested as frequent false peaks and valleys, seriously interfering with the accuracy of step detection. Hence, it is necessary to denoise the acceleration modulus data first. The commonly used smartphones can directly output the three-axis acceleration $a_{t}^{x}$, $a_{t}^{y}$, and $a_{t}^{z}$ without eliminating the influence of gravity. The acceleration modulus is calculated as follows:

(2) $$a_{t}=\sqrt{{\left(a_{t}^{x}\right)}^{2}+{\left(a_{t}^{y}\right)}^{2}+{\left(a_{t}^{z}\right)}^{2}}$$

**Figure 2:**
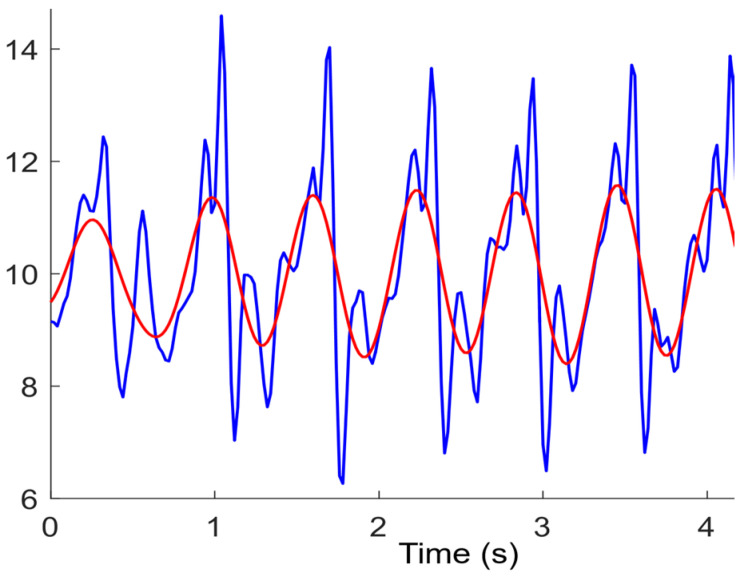
Comparison before (blue) and after (red) Butterworth denoising for acceleration modules.

In this work, we adopt the Butterworth filter, also known as the maximum flatness filter [23]. The characteristic of a Butterworth filter is that the frequency response curve within the passband is as flat as possible without ripple, while gradually decreasing to zero in the stopband. The cutoff frequency and order of the Butterworth filter are set to 0.08 and 4, respectively. Figure 2 shows the denoised results of the Butterworth filter. We can observe that the Butterworth filter significantly smoothes the waveform of the acceleration modulus and reduces the interference of noise.

Considering that the acceleration modulus will show periodic changes in peaks and valleys as the gait changes, steps can be counted by identifying the peaks and valleys of the acceleration modulus. The step identification method for wave peaks and wave valleys is as follows [24]:

(3) $$\left\{\begin{array}{l} \mathrm{peak}=\{\;t\;|a_{t,low}>a_{t-1,low}\;\&\&\;a_{t,low}>a_{t+1,low}\}\\ \mathrm{vally}=\{\;t\;|a_{t,low}<a_{t-1,low}\;\&\&\;a_{t,low}<a_{t+1,low}\} \end{array}\right.$$

where *a_t,low_* is obtained by passing *a_t_* through the Butterworth filter.

We conduct a step detection experiment. The tester held a smartphone and walked 50 steps, 80 steps, and 100 steps at three speeds: slow, normal, and fast. Before walking, the tester held a smartphone and stood still for about 5 s. After walking, the tester also stayed still for about 5 s to analyze the complete walking cycle. Table 1 lists the statistical results of the step detection. The average success rate of identifying steps using the peak-valley method is above 96% for most of the cases, indicating that the detection accuracy is ideal.

### 2.3. Step Length Estimation

Step length is affected by many factors: (1) physical characteristics, such as height and weight; (2) road characteristics, such as uphill slopes and turns; (3) the pedestrian’s acceleration during walking. Usually, the step lengths of different pedestrians are different. Even the same pedestrian’s step lengths are not necessarily the same in different motion states. Therefore, the step length estimation model should not be related to the specific pedestrian, and should not include the pedestrian’s body characteristic parameters [25]. In this paper, we adopt the Weinberg model [26], which is a nonlinear model built using acceleration parameters:

(4) $$SL=K_{1}\cdot \sqrt[{4}]{a_{\max}-a_{\min}}$$

where *K*_1_ is empirically taken as 0.364 based on our previous tests; *a*_max_ and *a*_min_ are the extreme values of acceleration in a gait cycle.

In the step length estimation experiment, the tester held a smartphone and walked 50 steps, 80 steps, and 100 steps at different speeds: slow, normal, and fast. The actual walking distance and the estimation results of the Weinberg model are shown in Table 2. It can be seen that with an average walking distance of 48.3 m each time, the average error of the Weinberg model is only 1.553 m, so the step length estimation is relatively accurate.

### 2.4. Heading Estimation

In PDR, accurate estimation of the pedestrian’s heading is crucial to the final positioning result. We use the Mahony algorithm-based complementary filter [27,28] and assume that the acceleration data in the body frame is ***a****_b_*, the magnetometer output is ***m****_b_*, and the gyroscope output is ***w****_b_*. We choose the geometric center of the phone as the center of the body frame. When the phone is placed horizontally, the right side is the x-axis, the forward side is the y-axis, and the z-axis conforms to the right-hand system. We first normalize the acceleration and magnetometer data using their 3D modulus:

(5) $$\left\{\begin{array}{l} {\hat{a}}_{b}={\left[\begin{array}{ccc} a_{x}/\sqrt{a_{x}^{2}+a_{y}^{2}+a_{z}^{2}} & a_{y}/\sqrt{a_{x}^{2}+a_{y}^{2}+a_{z}^{2}} & a_{z}/\sqrt{a_{x}^{2}+a_{y}^{2}+a_{z}^{2}} \end{array}\right]}^{T}\\ {\hat{m}}_{b}={\left[\begin{array}{ccc} m_{x}/\sqrt{m_{x}^{2}+m_{y}^{2}+m_{z}^{2}} & m_{y}/\sqrt{m_{x}^{2}+m_{y}^{2}+m_{z}^{2}} & m_{z}/\sqrt{m_{x}^{2}+m_{y}^{2}+m_{z}^{2}} \end{array}\right]}^{T} \end{array}\right.$$

where ${\left[\begin{array}{ccc} a_{x} & a_{y} & a_{z} \end{array}\right]}^{T}$ and ${\left[\begin{array}{ccc} m_{x} & m_{y} & m_{z} \end{array}\right]}^{T}$ are the readings of the accelerometer and magnetometer under the body frame, respectively. We assume that the gravity acceleration in the navigation frame is $\tilde{g}$, which can be expressed as ***g****_n_* = [0 0 1]^T^ after normalization. As for the navigation frame, we still choose the geometric center of the mobile phone as the center, the local east direction as the x-axis, the direction as the y-axis, and the vertical upward axis as the z-axis. That is, the navigation frame coincides with the local east–north–up (ENU) coordinate system. After coordinate transformation [29], the theoretical acceleration ***v*** in the body frame can be obtained. Based on this, the correction vector ***e****_a_* of the acceleration error can be further obtained:

(6) $$\left\{\begin{array}{l} v=C_{n}^{b}g_{n}\\ e_{a}={\hat{a}}_{b}⊗v \end{array}\right.$$

where $C_{n}^{b}$ is the rotation matrix from navigation frame to the body frame, ⊗ represents the external product operator, and ***v*** represents the gravity acceleration in the body frame. Convert the normalized magnetometer data from the body frame to the navigation frame:

(7) $$h=C_{b}^{n}{\hat{m}}_{b}={\left[\begin{array}{ccc} h_{x} & h_{y} & h_{z} \end{array}\right]}^{T}$$

where h are the magnetometer data under the navigation frame, and $C_{b}^{n}$ is the rotation matrix from the body frame to the navigation frame. In addition, $C_{b}^{n}={\left(C_{n}^{b}\right)}^{T}$. In the east–north–up (ENU) coordinate system, the theoretical magnetic field strength is

(8) $$l={\left[0\;\sqrt{h_{x}^{2}+h_{y}^{2}}\;h_{z}\right]}^{T}$$

From this, the theoretical output ***u*** of the magnetometer in the body frame can be calculated, and the magnetic error correction vector ***e****_m_* can be obtained:

(9) $$\left\{\begin{array}{l} u=C_{n}^{b}l\\ e_{m}={\hat{m}}_{b}⊗u \end{array}\right.$$

Based on the acceleration error correction vector ***e****_a_* in Equation (6) and the magnetic error correction vector ***e****_m_* in Equation (9), the gyroscope error correction vector ***δ*** and gyroscope data correction can be obtained:

(10) $$\left\{\begin{array}{l} \delta =K_{P}(e_{a}+e_{m})+K_{I}\int \left(e_{a}+e_{m}\right)\\ w=w_{b}+\delta \end{array}\right.$$

where *K_P_* and *K_I_* are predefined constants, and w is the corrected gyroscope data. After this correction, the attitude can be updated according to the quaternion algorithm.

**Figure 3:**
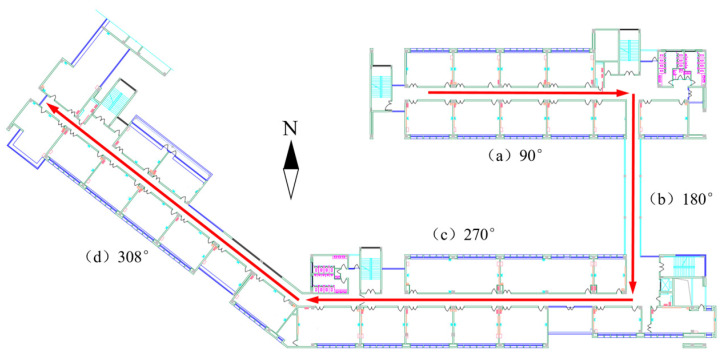
The walking route at the School of Environment and Spatial Informatics, China University of Mining and Technology.

We tested the reliability of the Mahony algorithm at the School of Environment and Spatial Informatics (SESI), China University of Mining and Technology (CUMT). Before walking, the tester first stood still for 5 s, and then walked the entire journey at a constant speed. The walking trajectory of the experiment is shown in Figure 3, in which the reference directions of sections a, b, c, and d are 90°, 180°, 270°, and 308°, respectively. Table 3 shows the headings of each road section estimated by the Mahony algorithm. We observe that the Mahony algorithm accurately estimates the heading of each road section. The average heading estimation error of the four road sections is only 5.25°.

## 3. GNSS/PDR Fusion Positioning

**Figure 4:**
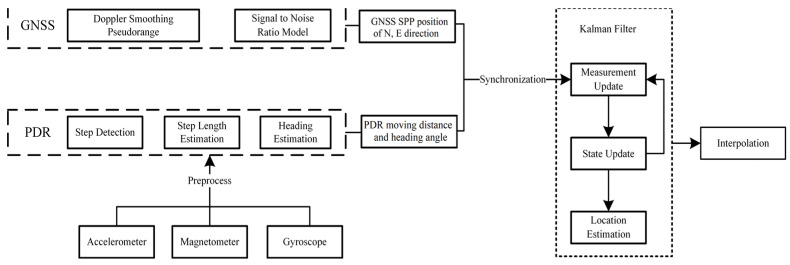
Flowchart of GNSS/PDR fusion positioning.

GNSS can provide high-precision absolute position information, but is limited to a scenario with unblocked signals. In a scenario with blocked GNSS signals and a severe multipath effect, the positioning accuracy will significantly degrade by the decrease in received satellite numbers and the deterioration of observation quality. PDR provides relative location information. Although its positioning accuracy is not affected by external factors, its positioning errors always accumulate over time. In this section, we combine GNSS with PDR for fusion positioning. As shown in Figure 4, by combining the absolute position information of GNSS and the relative position information of PDR, the positioning advantages of different technologies are integrated to improve positioning accuracy and robustness. The Doppler smoothed pseudorange [30,31], combined with the signal-to-noise ratio model, is used to provide the horizontal position with single point positioning mode, and then input as part of the measurement update. The PDR output is another part of the measurement update.

### 3.1. GNSS/PDR Fusion Positioning Algorithm

In GNSS/PDR fusion positioning, the horizontal position coordinates (*E*, *N*) and course angle *ψ* of the smartphone are taken as the state vector of the Kalman filter, and then the prediction update can be expressed as

(11) $$\left\{\begin{array}{l} E_{k}=E_{k-1}+l_{k}\cdot \sin\psi_{k}+\omega_{E}\\ N_{k}=N_{k-1}+l_{k}\cdot \cos\psi_{k}+\omega_{N}\\ \psi_{k}=\psi_{k-1}+\omega_{\psi } \end{array}\right.$$

where, *ω_E_*, *ω_N_*, and *ω_ψ_* are denoted as system noise. The state transition matrix ***F****_k_*_/*k*−1_ and system noise matrix ***Q****_k_*_−1_ shown in the Equation (11) are expressed as

(12) $$F_{k/k-1}=\left[\begin{array}{ccc} 1 & 0 & l_{k}\cos\psi_{k}\\ 0 & 1 & -l_{k}\sin\psi_{k}\\ 0 & 0 & 1 \end{array}\right]$$

(13) $$Q_{k-1}=\left[\begin{array}{ccc} \sigma_{E}^{2} & 0 & 0\\ 0 & \sigma_{N}^{2} & 0\\ 0 & 0 & \sigma_{\psi }^{2} \end{array}\right]$$

The measurement vector includes the GNSS horizontal coordinates (*N*, *E* components) and PDR heading angle after coordinate conversion. Measurement update is expressed as

(14) $$\left\{\begin{array}{l} E_{k}^{z}=E_{k}+v_{E^{z}}\\ N_{k}^{z}=N_{k}+v_{N^{z}}\\ \psi_{k}^{z}=\psi_{k}+v_{\psi^{z}} \end{array}\right.$$

where $v_{E^{z}}$, $v_{N^{z}}$, and $v_{\psi^{z}}$ represent measurement noise. The measurement matrix ***H****_k_* and the measurement noise matrix ***R****_k_* shown in Equation (13) are expressed as

(15) $$H_{k}=\left[\begin{array}{ccc} 1 & 0 & 0\\ 0 & 1 & 0\\ 0 & 0 & 1 \end{array}\right]$$

(16) $$R_{k}=\left[\begin{array}{ccc} \sigma_{E^{z}}^{2} & 0 & 0\\ 0 & \sigma_{N^{z}}^{2} & 0\\ 0 & 0 & \sigma_{\psi^{z}}^{2} \end{array}\right]$$

In Section 3.2, Section 3.3 and Section 3.4, we will evaluate the performance of GNSS/PDR fusion positioning in an open scenario, a semiopen scenario, and a blocked scenario.

### 3.2. Positioning Performance in the Open Scenario

**Figure 5:**
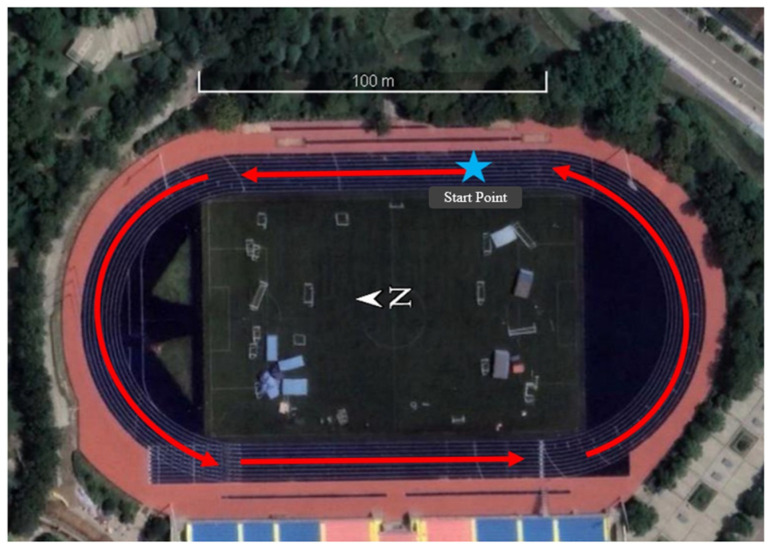
Trajectory of GNSS/PDR data acquisition under the open scenario.

The first sports field of Nanhu Campus at CUMT was selected for the open scenario tests. The data were collected on 13 December 2022 at 2:00 p.m. As shown in Figure 5, the tester walked two circles around the sports field, which took about 14 min. The data acquisition equipment was a Huawei P40 smartphone and two Hi-Target iRTK5 GNSS receivers, one of which moved synchronously with the smartphone as a mobile station, and the other receiver was set up on the rooftop of SESI at CUMT as a reference station. The Huawei P40 smartphone can support receiving dual-frequency and multiconstellation GNSS observations; however, only one frequency of each constellation was used in the subsequent GNSS SPP resolution. GNSS data were sampled at 1 Hz for both receivers, and inertial information was sampled at 100 Hz on the smartphone. In the whole observation period, the average number of received satellites per epoch of the mobile phone was 34, and the average GDOP value was 1.441. The real-time kinematic (RTK) results of the GNSS receiver were used as the reference for the GNSS/PDR fusion positioning.

**Figure 6:**
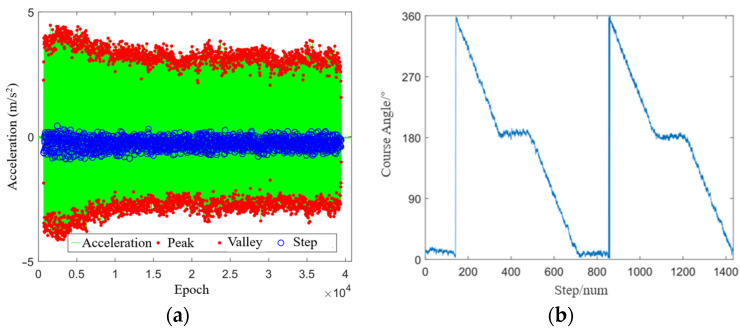
Experiment results under the open scenario: (**a**) step detection; (**b**) heading estimation.

Figure 6a shows the step detection results in the open scenario. It can be seen that the peak–valley step detection method identified 1432 steps, and the tester walked at a constant speed during the test period. Figure 6b shows the heading estimation results of the Mahony algorithm. It can be seen that the tester first walked northwards with a course angle of about 10°, then turned around at a constant speed and walked southwards with a course angle of about 185°, and finally returned to the starting point and repeated the action. The results of heading estimation are in complete agreement with reality, which verifies the reliability of the Mahony algorithm.

**Figure 7:**
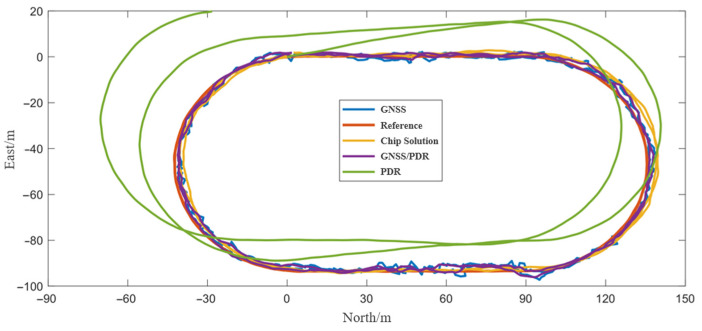
Trajectories of different methods under the open scenario.

Figure 7 shows the positioning trajectories of GNSS, PDR, GNSS/PDR, and smartphone chip solutions in the open scenario. It can be seen that the PDR error was gradually increasing with the increasing walking time. Additionally, compared with the results of GNSS and chip solutions, the trajectory of GNSS/PDR fusion positioning was smoother and more accurate. Table 4 shows the positioning accuracy statistics of the compared schemes. Compared with the root mean square error (RMSE) of GNSS and chip solution, the RMSE of GNSS/PDR fusion positioning decreased by 7% and 31%, respectively. Moreover, the error statistical indicators such as mean and maximum, also show that the fusion positioning algorithm had the highest positioning accuracy, indicating the advantages of fusion positioning.

### 3.3. Positioning Performance in the Semiopen Scenario

**Figure 8:**
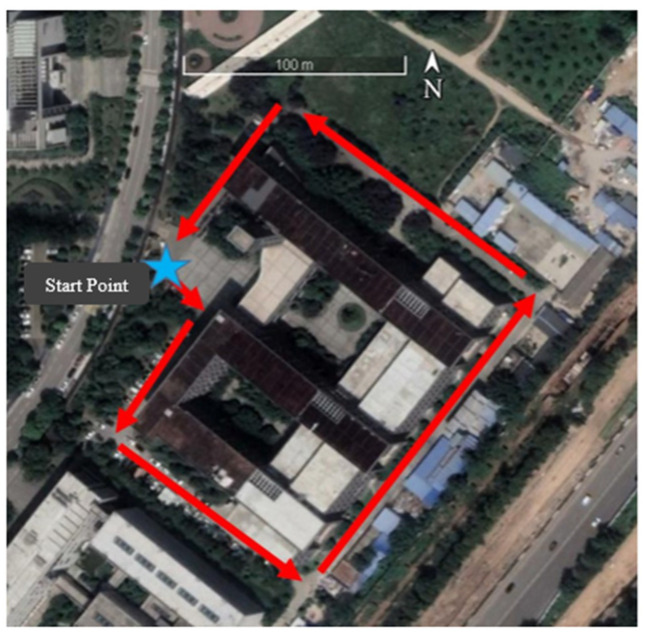
The trajectory of GNSS/PDR data acquisition in the semiopen scenario.

The Mining Science Center (MSC) of CUMT was selected as the semiopen scenario, and the data were collected on 13 December 2022, at 3:00 p.m. The test scenario and trajectory are shown in Figure 8. The volunteer walked around the MSC at a constant speed, which took about 8 min, and the other settings of the experiment were the same as above. In the whole observation period, the average number of received satellites per epoch of the smartphone was 32, and the average GDOP value was 2.317. Compared with the open scenario, the rise in GDOP is because the satellite signal was partially blocked by obstacles.

**Figure 9:**
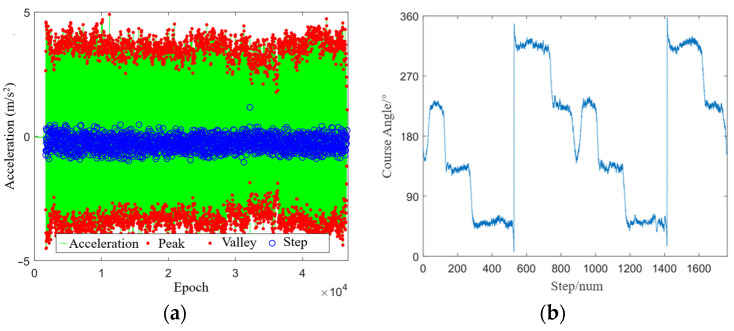
Experiment results under the semiopen scenario: (**a**) step detection; (**b**) heading estimation.

**Figure 10:**
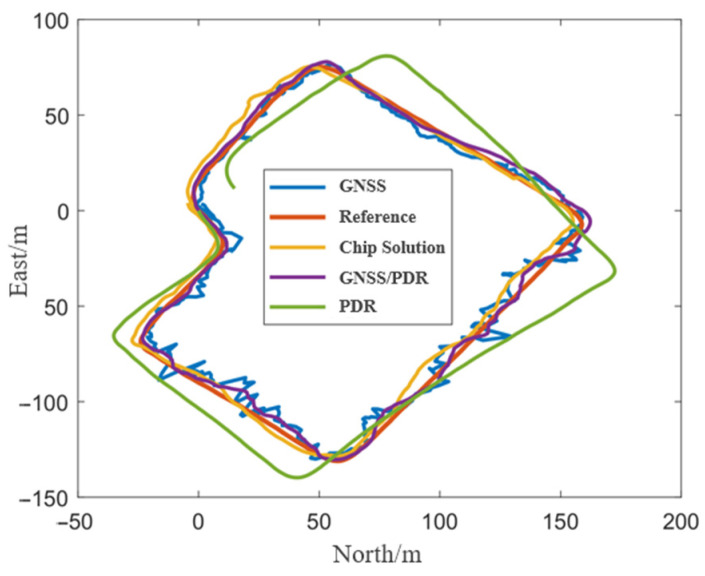
Trajectories of different methods under the semiopen scenario.

Figure 9 shows the results of step detection and heading estimation under the semiopen scenario. In this experiment, the actual number of steps was 920, and the result of step detection was 899. The detection success rate was 97.7%. Figure 10 shows the positioning trajectories of different methods. It can be seen that the positioning trajectory of PDR in the initial straight stage was consistent with the reference trajectory, but the trajectory was biased after the first turn, and then the positioning error was accumulated continually. And finally, the starting point and end point could not be matched. In some epochs, GNSS positioning results deviated from the reference. However, the GNSS/PDR fusion positioning improved the performance, and its positioning trajectory is in good agreement with the reference. Table 5 shows the positioning accuracy statistics of different methods. The RMSE of GNSS/PDR fusion positioning was decreased by 27% and 29%, respectively, in comparison with GNSS and chip solutions, which verifies the advantages of fusion positioning again.

### 3.4. Positioning Performance under the Blocked Scenario

**Figure 11:**
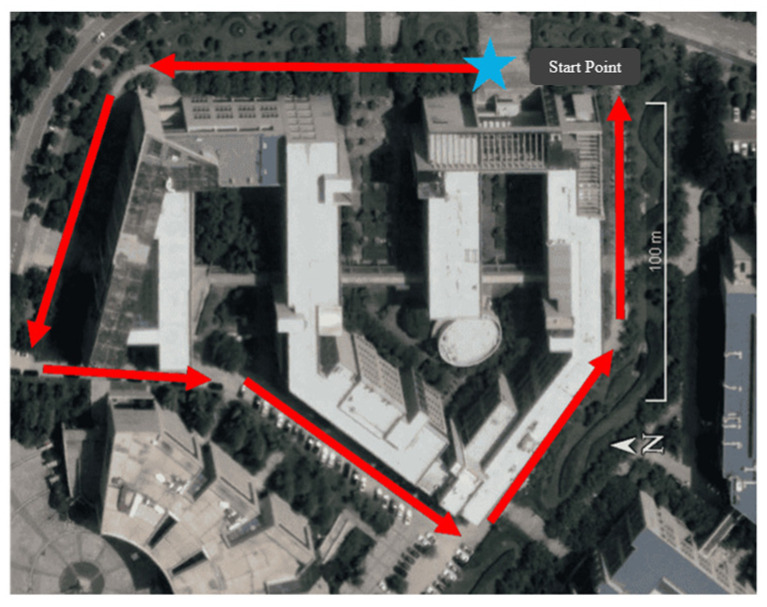
Trajectory of GNSS/PDR data acquisition under the blocked scenario.

The SESI was selected as the blocked scenario, and the data were collected on 13 December 2022, at 4:00 p.m. The test scenario and trajectory are shown in Figure 11. In the whole observation period, GNSS signals were mostly blocked by trees and buildings. The average number of received satellites per epoch of the mobile phone was 31, resulting in an average GDOP value of 2.635.

**Figure 12:**
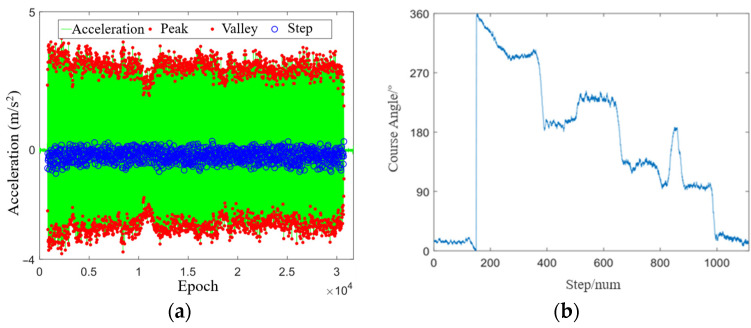
Experiment results under the blocked scenario: (**a**) step frequency detection; (**b**) heading estimation.

**Figure 13:**
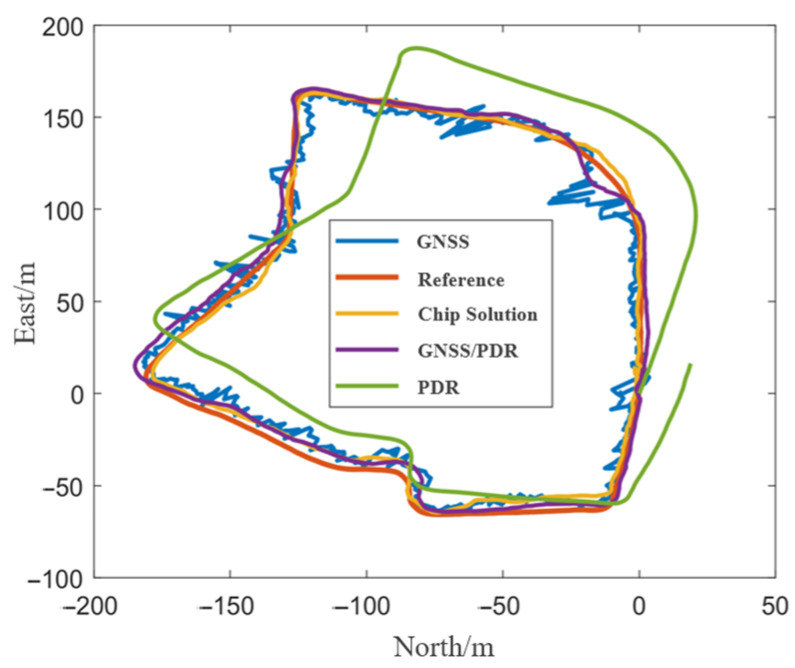
Trajectories of different methods under the blocked scenario.

Figure 12 shows the results of step detection and heading estimation under the blocked scenario. In the experiment, the actual number of steps was 1130, the result of step detection was 1110, and the detection success rate was 98.0%. Figure 13 shows the trajectories of different methods under the blocked scenario. Compared with the open scenario and the semiopen scenario, the GNSS positioning performance in the blocked scenario was degraded. Additionally, some positioning results contain serious bias, while the GNSS/PDR fusion positioning smoothed the abnormal GNSS positioning results and significantly improved the positioning performance. Table 6 lists the positioning accuracy of different methods. It can be seen that the RMSE of GNSS/PDR under the blocked scenario was decreased by 33% and 13%, respectively, compared with that of GNSS and chip solution, which also verifies the advantages of multisensor fusion positioning under the complex scenario.

## 4. Development and Performance Analysis of the Real-Time Fusion Positioning Software

Based on the former analysis, we verified the effectiveness and reliability of the GNSS/PDR fusion positioning on smartphones through post-processing and found that the fusion positioning algorithm helps improve positioning accuracy in challenging scenarios. Considering that post-processing usually cannot meet human positioning requirements, we developed a real-time fusion positioning software, called CUMT-POS version 1.0, on smartphones’ Android system. In the following, we will introduce the design structure, functions, and positioning performance of the software, respectively.

### 4.1. Design Framework and Functions

**Figure 14:**
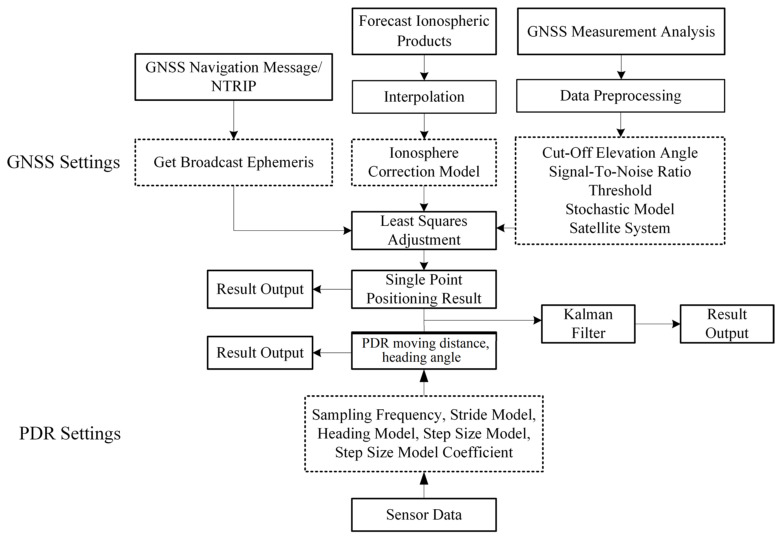
Flowchart of GNSS/PDR real-time positioning algorithm.

The flowchart of GNSS/PDR real-time fusion positioning software is shown in Figure 14. Readers may find the link between Figure 14 and Figure 4, that is, Figure 14 provides the actual real-time implementation of the conceptual framework in Figure 4. With raw GNSS measurements collected by smartphones, the users can freely adjust some parameter configurations conveniently for their own positioning needs, such as cutoff elevation angle, signal-to-noise threshold, defining the stochastic model, and choosing which constellation to use. Combined the preprocessed GNSS measurements with ionospheric correction and broadcast ephemeris, one can readily obtain the GNSS single point positioning (SPP) result. In terms of software development and testing environment, we selected the Android Studio compiler, and the development language is JDK 11 Java. Since Google only opened the interface for raw GNSS data output in Android 7.0 (corresponding to API 24) and above, our fused positioning software is not suitable for those smartphones below Android 7.0. The smartphone we used for software testing is the Xiaomi 8, equipped with Android 10 (corresponding to API 29). The smartphone supports receiving dual-frequency and multiple-constellation GNSS observations, including GPS, GLONASS, BDS, Galileo, and QZSS.

**Figure 15:**
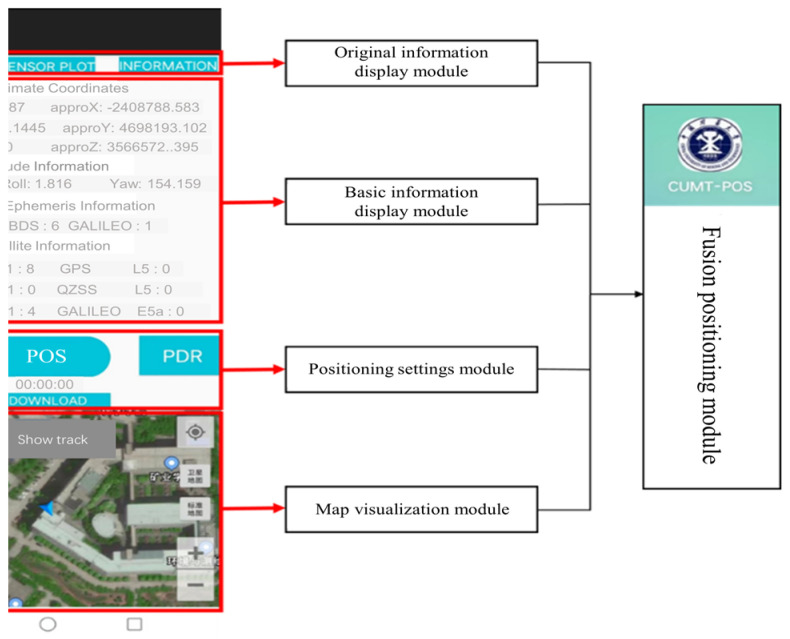
Software architecture of GNSS/PDR real-time fusion positioning.

Figure 15 shows the four major modules of CUMT-POS version 1.0, including the original information display module, the basic information display module, the positioning setting module, and the map visualization module. The four modules are introduced as follows:

(1) Original information display module

**Figure 16:**
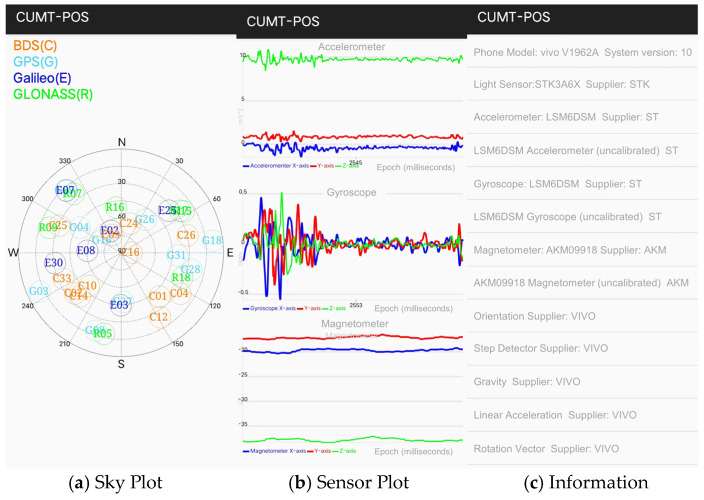
Original information display module.

In this module, users can quickly learn the current status of GNSS data and PDR data, and draw starry sky maps, PDR data time series, and smartphone sensor lists, respectively, as shown in Figure 16.

(2) Basic information display module

**Figure 17:**
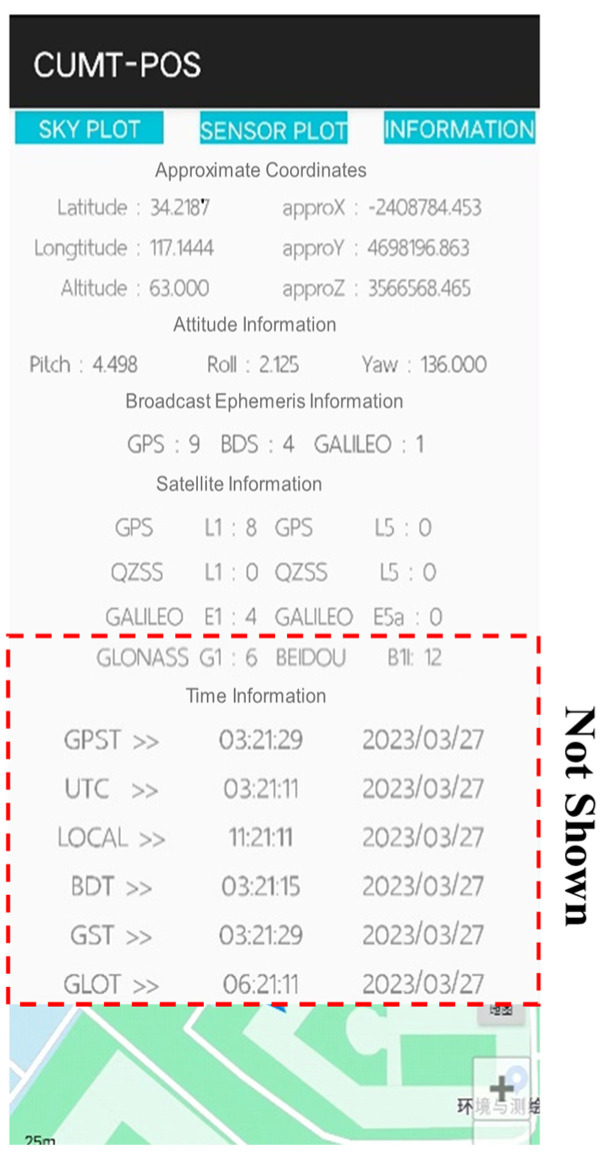
Basic information display module.

In this module, users can learn the initialization status of GNSS and PDR data, including the approximate coordinates of the user’s current location, the attitude of the smartphone, the acquisition of broadcast ephemeris, the number of GNSS single- and dual-frequency observation data, and time system conversion. In addition, the window of this module is sliding. Users can browse the basic information that cannot be fully displayed by sliding up and down, as shown in Figure 17.

(3) Map visualization module

The map visualization module is based on AMAP SDK. This module has functions such as trajectory display, map layer switching, location tracking, and map zooming.

(4) Positioning setting module

**Figure 18:**
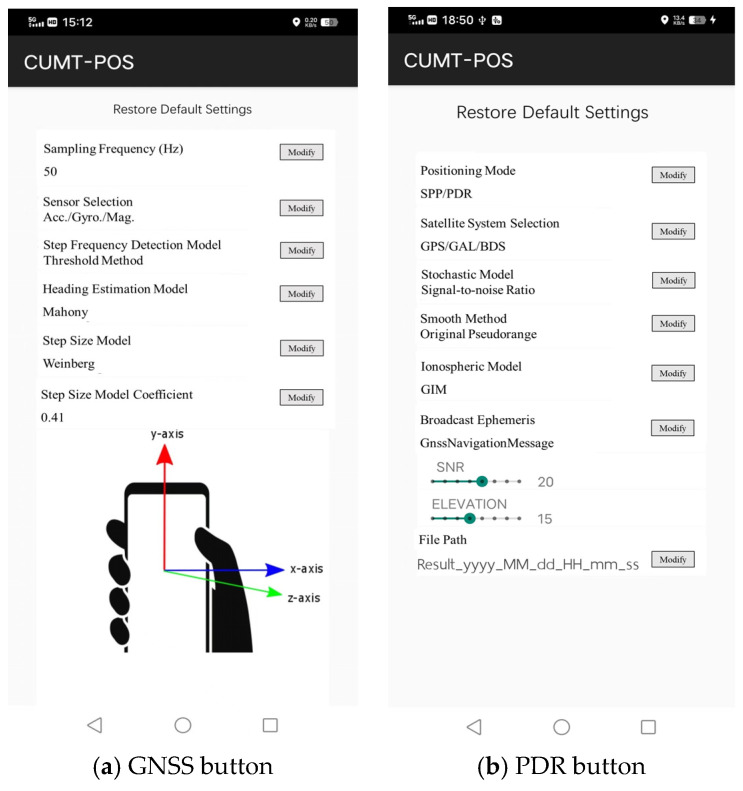
Positioning settings module.

The positioning setting module is the core module of CUMT-POS version 1.0, including four buttons and one positioning timer. First, clicking the “GNSS” button will open the GNSS positioning parameter setting interface. Users can select the positioning model and set parameters as needed. For example, in the “Positioning Model” setting, users can select modes such as SPP, PDR, SPP/PDR, etc., as shown in Figure 18a. Second, clicking the “PDR” button will open the PDR parameter-setting interface. Users can set the sampling frequency as needed and select the algorithm for heading estimation, step length estimation, and step detection, as shown in Figure 18b. Third, the “Position” button is used to configure the startup and shutdown of fusion positioning. When it is on, the button will turn red. Fourth, by clicking the “DOWNLOAD” button, one can then download the 5 min updated real-time global ionospheric modeling (GIM) correction products from ftp://igs.gnsswhu.cn/pub/whu/MGEX/realtime-ionex/ (accessed on 10 February 2023).

After configuring the four major modules of CUMT-POS version 1.0, the final positioning solution will directly output to the smartphone storage space as a new file.

### 4.2. GNSS/PDR Real-Time Positioning Software Performance Analysis

**Figure 19:**
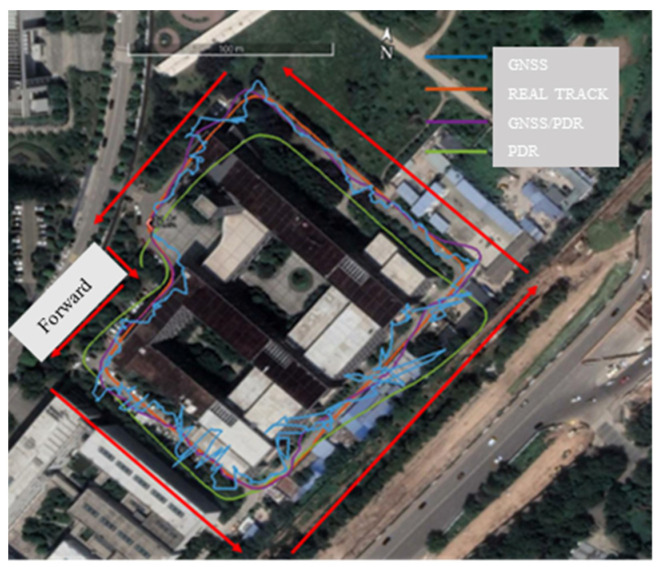
Motion trajectories solved by different positioning algorithms: compared with the real trajectory.

To test the effectiveness of CUMT-POS version 1.0, we chose MSC as the experimental site. The data were collected on 14 December 2022, at 9:00 a.m. We set up the GNSS base station on the sixth-floor rooftop of SESI, with a sampling frequency of 1 Hz, and the receiver RTK solutions were taken as reference. The positioning trajectories of different algorithms are shown in Figure 19. When only using GNSS or only using PDR, the positioning error was large, and in many epochs, the positioning solutions deviated from the true trajectory. In short, the accuracy of GNSS/PDR fusion positioning was significantly higher than that of only-GNSS solution and only-PDR solution.

Table 7 summarizes the accuracy statistics of GNSS and GNSS/PDR positioning using the developed real-time fusion positioning software CUMT-POS version 1.0. Compared with the GNSS algorithm, the positioning accuracy of the GNSS/PDR algorithm improved by 26%. In addition, the fusion positioning algorithm has more advantages in terms of mean, maximum, and median positioning errors. This shows that the fusion algorithm has better robustness and stability than a single positioning algorithm.

## 5. Conclusions

In this paper, we first studied the peak–valley step detection algorithm, the Weinberg step length estimation algorithm, and the Mahony heading estimation algorithm in PDR. Then, we analyzed the positioning performance of the GNSS/PDR fusion algorithm in an open environment, semiopen environment, and blocked environment, respectively. Finally, we developed a real-time GNSS/PDR fusion positioning software, called CUMT-POS version 1.0, suitable for smartphones running Android 7.0 and above. Our experimental results show the following: (1) In terms of step detection, the accuracy of the peak–valley detection algorithm is ideal, and the detection success rate is 99.13% in the slow walking state, 98.70% in the common walking state, and 94.78% in the fast walking state. The average error of the Weinberg model is only 1.553 m at an average distance of 48.3 m, and the heading estimation by the Mahony algorithm is also consistent with reality. (2) In all three kinds of scenarios with different levels of occlusion, GNSS/PDR fusion shows significant improvements in positioning accuracy and stability, compared with only GNSS, only PDR, and smartphone chip solutions. (3) After evaluating the positioning performance of CUMT-POS version 1.0, we conclude that the horizontal positioning accuracy of the developed software is improved by 26% compared with only GNSS, which again verifies the effectiveness of real-time fusion positioning software.

## Figures and Tables

**Table 1:** Step detection results of the peak–valley method.

Real Steps	Speed	Step Count	Accuracy
50	Slow	50	100.00%
	Normal	51	98.00%
	Fast	52	96.00%
80	Slow	81	98.75%
	Normal	81	98.75%
	Fast	73	91.25%
100	Slow	101	99.00%
	Normal	101	99.00%
	Fast	97	97.00%

**Table 2:** Step length estimation results of the Weinberg model.

Speed	Real Distance/m	Estimated Distance/m	Distance Error/m
Slow	27.00	30.32	−3.32
	43.50	46.83	−3.33
	58.07	58.08	−0.01
Normal	32.40	31.51	0.89
	52.10	51.79	0.31
	65.57	61.72	3.85
Fast	35.40	34.79	0.61
	54.20	53.44	0.76
	66.17	65.27	0.90

**Table 3:** Estimated heading angle by the Mahony algorithm (°).

Algorithm	Section a	Section b	Section c	Section d
Reference value	90.00	180.00	270.00	308.00
Mahony algorithm	98.11	186.15	269.36	304.78

**Table 4:** Positioning accuracy statistics in the open scenario.

Method	RMSE/m	Mean/m	Maximum/m	Median/m
Chip Solution	3.455	3.263	6.228	3.198
GNSS	2.553	2.244	6.942	2.080
GNSS/PDR	2.385	2.078	6.040	2.011

**Table 5:** Positioning accuracy statistics in the semiopen scenario.

Method	RMSE/m	Mean/m	Maximum/m	Median/m
Chip Solution	4.957	4.418	9.125	4.600
GNSS	4.865	4.079	16.542	3.492
GNSS/PDR	3.535	3.072	8.002	2.963

**Table 6:** Positioning accuracy statistics in the blocked scenario.

Method	RMSE/m	Mean/m	Maximum/m	Median/m
Chip Solution	4.737	4.326	8.222	4.250
GNSS	6.089	4.962	27.733	4.016
GNSS/PDR	4.107	3.757	8.357	3.550

**Table 7:** Positioning error statistics of the developed software.

Positioning Method	RMSE/m	Mean/m	Maximum/m	Median/m
PDR	19.874	16.497	79.267	36.294
GNSS	9.756	7.295	57.015	5.158
GNSS/PDR	7.253	5.657	18.556	4.081

## Data Availability

Data are contained within the article.
